# Case report: Diagnosis and intervention of a non-24-h sleep–wake disorder in a sighted child with a psychiatric disorder

**DOI:** 10.3389/fpsyt.2023.1129153

**Published:** 2024-01-05

**Authors:** Carla Estivill-Domènech, Beatriz Rodriguez-Morilla, Eduard Estivill, Juan Antonio Madrid

**Affiliations:** ^1^Estivill-Sueño Foundation, Barcelona, Spain; ^2^Kronohealth, Murcia, Spain; ^3^Estivill Sleep Clinic, Barcelona, Spain; ^4^Chronobiology Lab, Department of Physiology, College of Biology, University of Murcia, IUIE, IMIB, Murcia, Spain

**Keywords:** sleep, circadian rhythms, N24SWD, free-running disorder, hypernycthemeral syndrome, psychiatric disorder

## Abstract

Circadian rhythm sleep–wake disorders (CRSWD) are sleep dysfunctions related to circadian functioning. They are characterized by symptoms of insomnia or excessive sleepiness that occur because the intrinsic circadian pacemaker is not entrained to a 24-h light/dark cycle. Affected individuals with a free-running disorder or hypernycthemeral syndrome (N24SWD) have a longer sleep–wake cycle that produces a sleep pattern that typically delays each day. The disorder is seen in 70% of blind people, and among people with healthy vision, it is a rare pathology. Among sighted cases, 80% are young men and 28% have a psychiatric disorder. The patient was a 14-year-old boy with a psychiatric pathology diagnosed with a PANDAS syndrome (pediatric autoimmune neuropsychiatric disorders associated with streptococci), a sudden acute and debilitating onset of intense anxiety and mood lability accompanied by obsessive compulsive-like issues and/or tics, in association with a streptococcal A infection that occurs immediately prior to the symptoms. As a comorbidity, he exhibited severe insomnia due to an irregular sleep pattern that strongly delayed his sleep schedule day to day. It affected his daily routines, as he was not going to school, and aggravated, furthermore, the psychiatric symptoms. He was referred for sleep consultation, where the case was explored by ambulatory circadian monitoring (ACM) using the novel system Kronowise^®^ (Chronolab, University of Murcia) and diagnosed with a non-24-h sleep–wake disorder (N24SWD). The first treatment approach for the patient was focused on improving symptoms during the acute infection and psychiatric symptoms. Additionally, sleep pathology was treated by light therapy and melatonin. After 8 months and different trials, it was possible to establish a treatment to normalize the symptoms and fix his sleep rhythm in a normal schedule as well as to reduce anxious symptoms during the day. The association of PANDAS and N24SWD has not previously been reported in the literature.

## Introduction

Sleep disorders are a different etiologic group of pathologies that are classified by the International Classification of Sleep Disorders (ICSD-3) into seven major categories (1). These pathologies cause insomnia, hypersomnolence, parasomnias, or movement disorders. This type of non-restorative or inadequate sleep impairs daytime functioning, emotional and mental wellbeing, physical functioning (2–4), and health (5–8).

Sleep disorders related to circadian functioning are the circadian rhythm sleep–wake disorders (CRSWD) (9, 10). Sleep/wake is a biological rhythm that repeats every 24 h, such as eating/fasting, body temperature, and many hormone secretions such as cortisol or melatonin. All these chronobiological functions that repeat every 24 h are called circadian rhythms. In humans, they are controlled by a central internal clock located in the suprachiasmatic nuclei of the hypothalamus (SCN), which acts as a pacemaker. Through molecular functioning by clock genes, the pacemaker produces oscillations and distributes them throughout the organism by means of ~10,000 neurons that communicate with other peripheral oscillators in order to synchronize metabolic, physiological, and behavioral rhythms, among others.

The correct human circadian system functioning must be fed by environmental cues, called zeitgebers, which act as synchronizers. As the internal clock rhythm is not exactly 24 h, these external cues are crucial to entrain with the natural 24-h cycle (11). The most important external synchronizer is the light/dark cycle. Photic and non-photic signals that the retina receives are delivered through the hypothalamic retinal pathway to reach the SCN. This oscillatory signal communicates, through various brain structures, with the pineal gland. This gland is responsible for synthesizing melatonin, which is the most important transmitter hormone of the circadian rhythm acting as the regulator of the sleep/wake rhythm, in the absence of light. On the contrary, when dawn begins to break and blue light hits the retina, the synthesis of this hormone stops (12).

CRSWD arises due to a misalignment between the sleep–wake rhythm and the natural 24-h cycle imposed by the Earth's rotation. This triggers symptoms, such as difficulty initiating and maintaining sleep, excessive sleepiness, and irregular sleep schedules, but also has an extended impact on social, occupational, and educational performance. The most common cause of circadian disorders is a non-entrained intrinsic circadian pacemaker because of social, psychological, or environmental factors. However, they can be also caused by alterations in the circadian time system or its entrainment mechanisms.

The most common circadian pathologies are the delayed sleep–wake phase disorder (DSWPD) and the advanced sleep–wake phase disorder (ASWPD), produced by a significant delay or advance, respectively, of the sleep phase in relation to the desired sleep–wake time. In the present article, the clinical discussion is related to a rare circadian disorder, the non-24-h sleep–wake rhythm disorder (N24SWD), also called a free-running disorder or hypernychthemeral syndrome. Individuals that suffer from this syndrome have an endogenous circadian period far from the 24-h light/dark cycle (13–15) that triggers a progressively delayed sleep–wake pattern producing the typical drift of the sleep need along the 24 h of the day. Symptoms depend on the period time of the asynchrony: individuals typically present insomnia, difficulties of sleep conciliation at night, excessive sleepiness, sleeping during the day at other times, or symptom remission when the sleep propensity coincides with the desired bedtime. These irregular schedules disrupt daytime functioning and socialization (16).

Melatonin prescribed to individuals suffering from N24SWD helps to improve their symptoms (17, 18), in addition to behavioral and environmental therapy to reinforce social rhythms in the treatment (19–22).

Non-24-h sleep–wake rhythm disorder is mainly associated with blind or visually impaired individuals, who have difficulty in photic/non-photic reception and therefore a lack of internal clock re-alignment (23).

Nevertheless, N24SWD affects individuals with intact vision in very rare cases. In these cases, it is postulated that the origin could come from irregular schedules and non-synchronizing social habits as well as from a defect in light reception due to poor perception. However, the extreme difficulty to entrain the circadian rhythm to a stable rhythm by means of zeitgebers suggests that the origin could be due to an alteration of the internal circadian system in the complex process of neuronal communication.

N24SWD in people with intact vision is more associated with young males, and up to 28% have a psychiatric disorder (24–26). Psychiatric disorders lead to an impairment of neurotransmitters, on which the sleep/wake rhythm is also dependent. Moreover, the abnormal social behaviors, anxiety, depression, and inappropriate exposure to environment and light, predispose them to a complex physiological-behavioral scenario for comorbid sleep disruption.

In general, sleep pathologies have an important comorbidity with psychiatric diseases (27, 28). As early as 1984, Tan, Kales, Soldatos, and Bixler determined that, out of 100 people attending the sleep specialist, two-third of them had some psychiatric disorder, and of these, half suffered from an affective disorder (29). The most common psychiatric causes of insomnia are psychosis, mood, anxiety, panic, and obsessive–compulsive disorder (OCD) as well as dementia (30–35).

Recent studies show exhaustive investigation of the relationship between psychiatric symptoms such as OCD or tics and the circadian system that controls the sleep/wake cycle (36, 37).

Pediatric neuropsychiatric disorders with an acute onset of anxiety and mood lability accompanied by obsessive–compulsive disorder (OCD) and/or tics exacerbated after a streptococcal infection are diagnosed specifically with pediatric autoimmune neuropsychiatric disorders associated with streptococci (PANDAS) (38–41). Even though the clinical presentations of OCD and tics are broadly related to a psychiatric cluster of symptoms, the comorbid sleep and circadian rhythm disruptions are not specifically described in the literature for PANDAS disease (42, 43).

This article presented the case of an adolescent diagnosed with a PANDAS syndrome who exhibited insomnia due to a severe irregular sleep pattern, which, in turn, aggravated psychiatric symptoms and triggered a social disruption. He was referred for sleep consultation. The diagnosis methodology and multidisciplinary treatment approach of the case are described.

## Patient information

The patient was a full-term infant born in 2003 by emergency cesarean section for cord prolapse and treatment of jaundice.

The parents reported that the patient had always had problems falling asleep. He woke up every 20–35 min with screaming, calming down spontaneously after 5 min. A sleep study was performed at the age of 2 years, without concluding any dysfunction. His sleep improved with strict hygiene habits, although he was always a light sleeper.

The patient was in a general good health, although with repeated episodes of pharyngitis in 2005 and nocturnal asthma in 2006, treated with montelukast and inhalers. In 2012, the patient tested positive for streptococcus and was treated with phenoxymethylpenicillin for 2 weeks. At the same time, learning changes were observed in mathematics, along with a change in handwriting style, which retrospectively was observed since 2005.

In August 2013, he moved to Sweden and quickly adapted to the new school. Suddenly, in November 2014, he began to have many fears and the first panic attacks when suggested to do activities involving peer relationships. At this time, sleep difficulties reappeared; sometimes he slept 6 h, while other nights he could not sleep at all. These anomalous patterns in his rest fed his fear of sleep, thus causing a lack of sleep that generated more irritation and anxiety.

In February 2015, the social fears worsened, and in May 2015, the patient suffered an anxiety attack in gym class and did not attend school again during that school year.

At the end of August 2015, the patient presented avoidance behaviors to enter his bedroom, delusions related to food contamination, and drastic changes in his manner, becoming aggressive. He also suffered from irrational fears and panic attacks on a daily basis, with fears such as sleeping alone. He began to present obsessions and compulsions with movements in the wrists and neck. The days when he did not sleep at all during the night were increasing, although he went back to sleep the following night.

In September 2015, his psychopathology was framed as a possible PANDAS, so he was admitted, and antibiotic treatment (penicillin) was started, rapidly improving the delusions and irrational fears. This diagnosis was under constant revision.

The psychiatric team treated the anxiety and sleep symptoms with antihistamines without achieving any improvement. The SSRIs, oxazepam and sertraline, caused increased anxiety and aggression. Melatonin at night (even at high doses, 4 mg) also elicited no response.

In November 2015, he was switched from antibiotics to amoxicillin, successfully eliminating obsessions and compulsions. Treatment was maintained along with probiotics, vitamin D, and magnesium.

The symptoms that led to the suspicion of PANDAS had subsided, but there were residual anxiety and learned anxiety due to the number of panic attacks he had suffered. For this reason, a psychological approach was proposed at that time, with the aim of socialization and his return to school.

In spite of everything, sleep dysfunction persisted. He displayed an irregular and unpredictable sleep rhythm; the patient's parents reported that he could sleep 2 h or 9 h and that, when he did not sleep during the night, over-excitation appeared during the day; then, he slept between 12 and 14 h in a row. Thus, in January 2016, he was referred to a sleep specialist in Barcelona. A virtual consultation was initiated with the patient's mother to take a medical history.

## Diagnostic assessment

The evaluation of sleep was addressed through a clinical history, a sleep diary, and an ambulatory circadian monitoring (ACM), as a diagnostic test. ACM consists of a multichannel device (Kronowise, Chronolab, University of Murcia) that is worn as a watch, which registers continuous recording of the body skin temperature (T), motor activity (A), body position (P), and exposure to light (L) of the subject for several consecutive days (44–46).

Sleep/wake rhythms are inferred using the integrated variable TAP using Circadianware^®^ software (Chronolab, University of Murcia). The analysis of the circadian variables was carried out using the average curves of all the recording days, during 24 h of the day. They are compared with curves that have been taken from a representative healthy population (47).

## Therapeutic intervention

The treatment of circadian rhythm disorders is based on forced guidance with external synchronizing agents, based on chronotherapy to set regular sleep times, light therapy to receive light at circadian times, and the administration of oral melatonin at the optimal chronobiological efficiency.

An adequate sleep schedule is planned in agreement for achieving correct sleep conciliation (10 p.m.−7 a.m.) and social needs. When his hypernycthemeral rhythm fell within this range, treatment would begin with guidelines that must be followed methodically: upon waking, light therapy was applied with a lamp followed by an outdoor exercise routine (bike ride); 4 h before going to sleep, he took melatonin (5 mg); during sunset and even before going to sleep, he should avoid using devices (avoid blue light from screens) and he used a mask during sleep, in case of natural light.

## Outcomes

**Figure 1:**
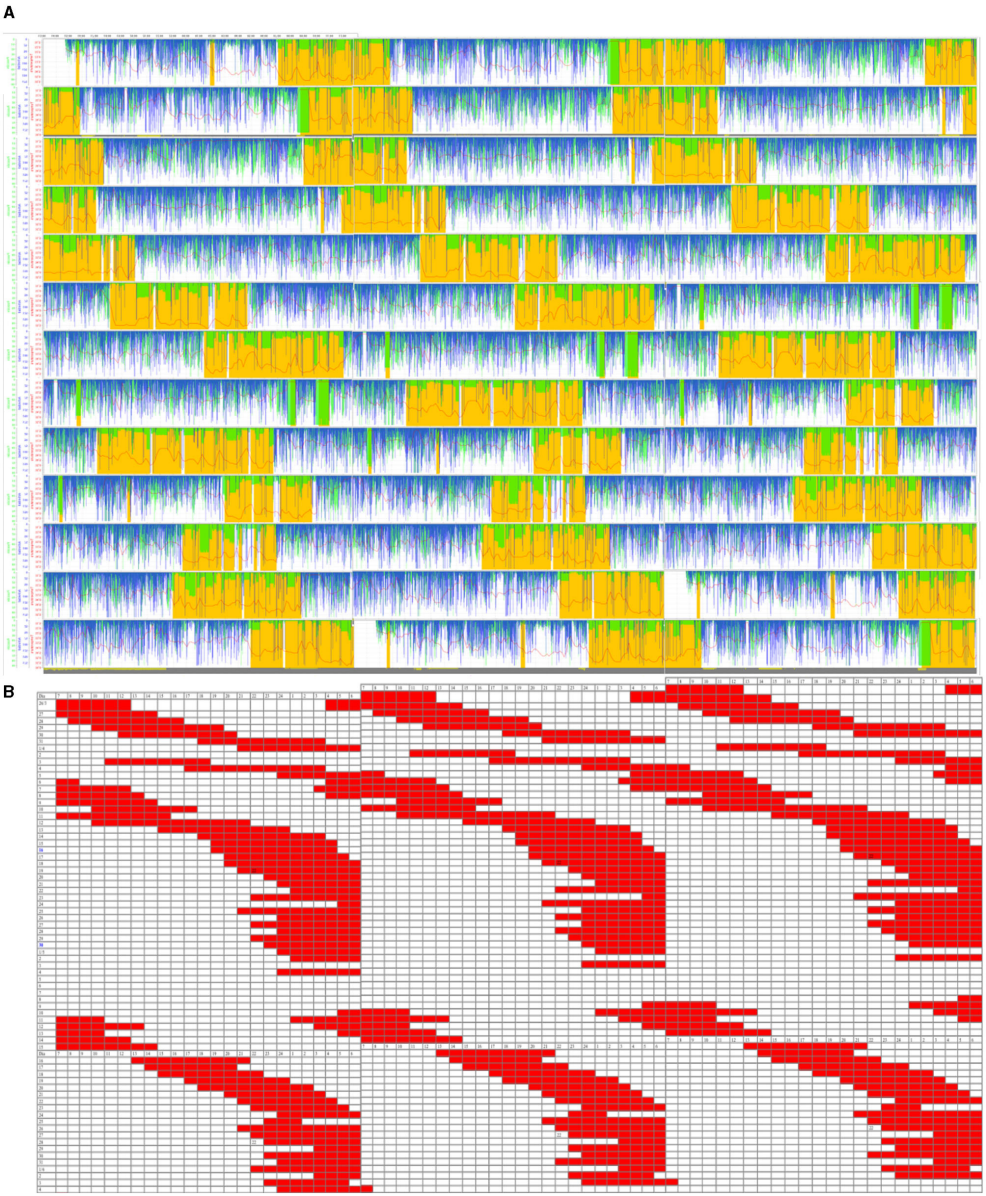
**(A)** Triple plot representation of the ambulatory circadian monitoring (ACM) daily recordings (13 days) of the patient. Variables are motor activity (min/h, in blue), body position (°, in green), and wrist temperature (°C, in red). Yellow-shaded areas are the sleep-predicted periods. **(B)** Triple plot representation of sleep periods (in red) noted in a diary (45 days). The blue band represents the night period.

From an ACM study for 13 days (see the triple plot in Figure 1A), the sleep periods were inferred, which showed a clear irregular sleep pattern outside the 24-h circadianity (N24SWD). At the same time, a sleep diary was made for 45 days (see the triple plot in Figure 1B), which showed that his sleep–wake cycle period was 25 h 16 min.

**Figure 2:**
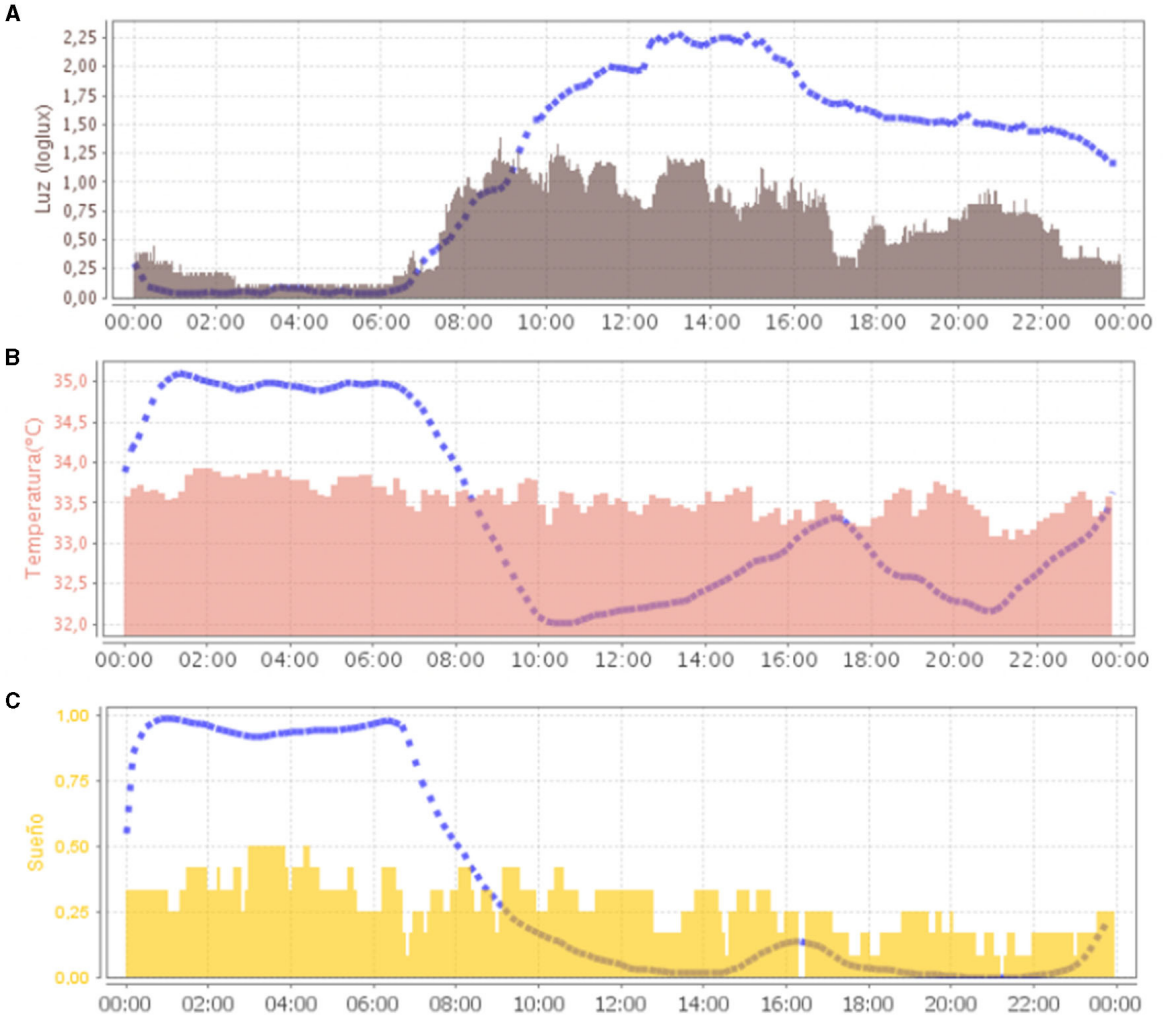
Representation (24 h in *X*-axis) of the mean waveform of light variable [**(A)**, in gray] and wrist temperature variable [**(B)**, in salmon) and sleep [**(C)**, in yellow]. Blue dashed lines represent the mean curve of a healthy group of reference.

The average representation of all recording days in ACM (Figure 2) showed a loss of circadianity of the patient's temperature variable, while normally it should be higher during night sleep. Regarding light, the greatest synchronizer of circadian rhythms, the patient's exposure during the day was also low, compared to normal values. This was mainly due to periods of sleep that occurred during the day.

Circadian sleep disorders occurred when there was a disruption of the internal pacemaker or a misalignment between sleep and the 24-h social and physical environment. In the case of the reported patient, desynchronization had occurred over a neurological and psychiatric alteration, which made a much more complex clinical scenario that aggravated symptoms. This non-24-h rhythm is aggravated by the complex geographic mobility of the family: the patient, originally from Spain, had lived in Sweden since 2013, which means that he had received different natural light hours from those he had received in Spain, and very different amounts of light between winter and summer.

## Results and patient perspective

**Figure 3:**
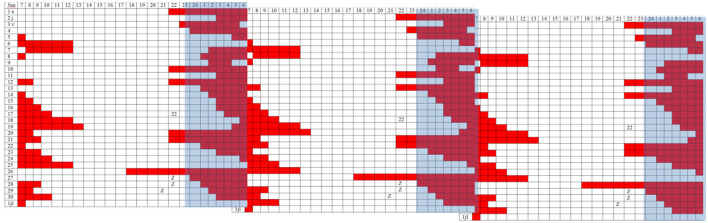
Triple plot representation of sleep periods (in red) noted in a diary (30 days). The blue band represents the night period.

In April 2016, treatment was started with synchronization of their circadian rhythms, forcing schedules with the help of all external synchronizers (light and melatonin). The forced guidance made it possible to establish a regular sleep schedule. However, there were days when the patient did not sleep at the correct time, and the sleep period drifted again (see log Figure 3), manifesting a strong internal resistance indicating that the synchronization strategy was not sufficient to restart the internal clock.

When the 24-h schedule was lost, the sleep period was left to drift back to the night, and therapy was started again. Psychological-behavioral treatment continued.

**Figure 4:**
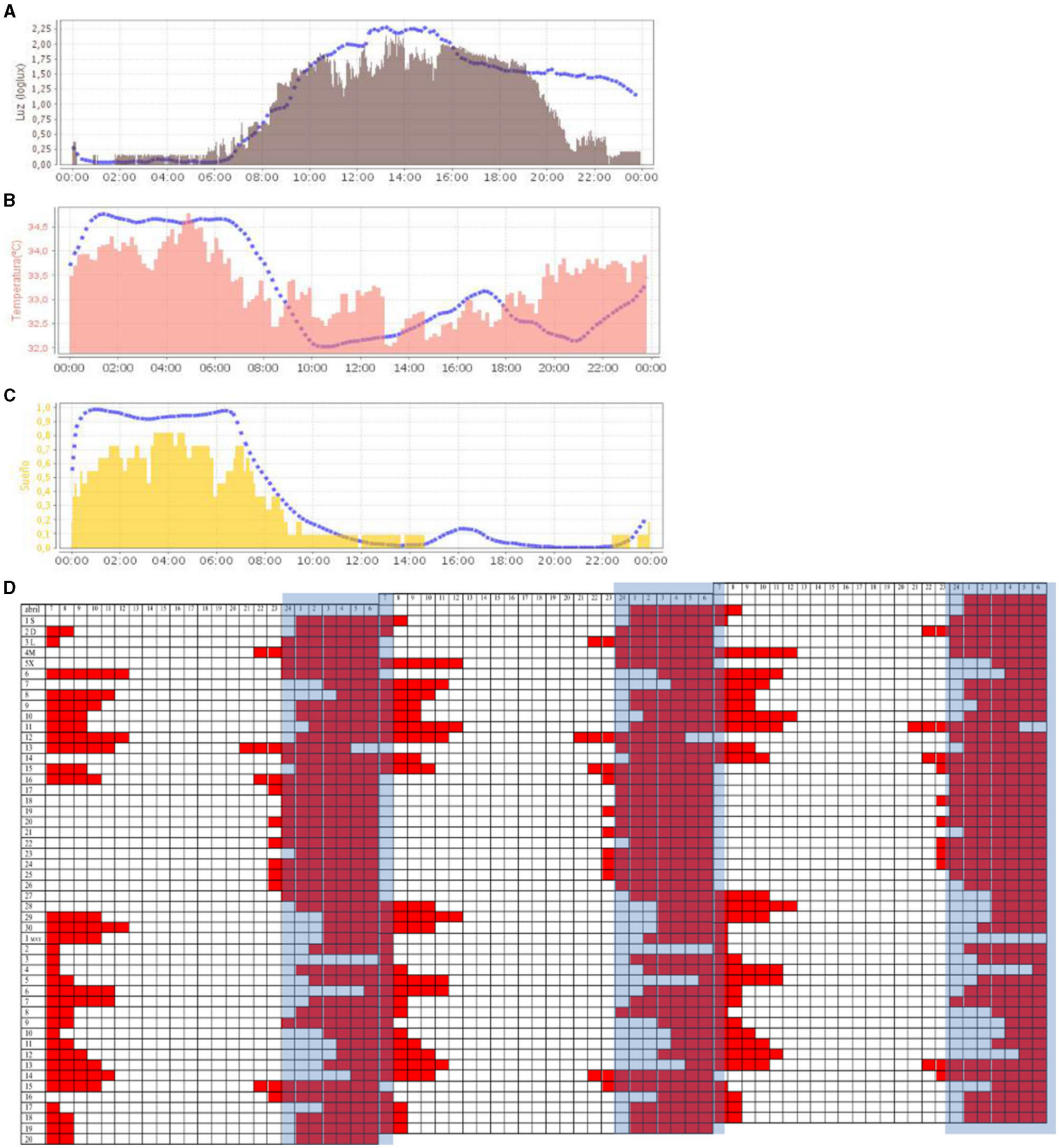
Representation (24 h in *X*-axis) of the mean waveform of light variable [**(A)**, in gray], wrist temperature variable [**(B)**, in salmon], and sleep [**(C)**, in yellow]. Blue dashed lines represent the mean curve of a healthy group of reference. [**(D)**] Triple plot representation of sleep periods (in red) noted in a diary (50 days). The blue band represents the night period.

At 6 months, the patient suffered a seizure, having been awake for 52 h. A neuroleptic antipsychotic (levomepromazine and aripiprazole) was administered, although this treatment caused drowsiness. After 3 months, the treatment was replaced by an anxiolytic (mirtazapine). At this time, a drastic improvement in the stabilization of sleep schedules could be observed, when resynchronization was applied again (see Figure 4). In the new ACM recording, the temperature, light, and sleep variables (Figures 4A–C) showed a great improvement: the alignment of habits was reflected in a synchronization of the light exposure curve, being high during the day and darkness contrasted by nighttime; body temperature shows the circadian contrast between day and night; the sleep period maintained a 24-h schedule centered on the night period.

With these combined guidelines, the patient's condition was optimal. He slept every night consecutively; the family felt that he was fresh and rested, he did not suffer from anxiety, his sociability and joy improved, his life normalized, and started attending school again.

## Discussion

In this report, we want to emphasize the importance of multidisciplinary care, referring the psychiatric case to the sleep unit. This has facilitated a differential diagnosis of sleep pathology. Often, all the multiple comorbid symptomatologies in a psychiatric dysfunction are non-specifically included in a global pharmacological treatment, being common the incorporation of hypnotics for sleep, which can be counterproductive for the rest of the daily symptoms. In addition, the method for the diagnosis confirms the great importance of actigraphy in the precision of detection of sleep pathologies and reinforces the need for its advancement, with new technologies with data as important as the temperature provided by ACM, used in the present case.

Finally, we would like to highlight the severe N24SWD dysfunction of the patient, as shown by the results, and its disabling effect on his daily functioning and on the psychiatric dysfunction associated with PANDAS. In his case, the treatment (light therapy, chronotherapy, and administration of melatonin) has demanded great persistence, time, and follow-up to finally achieve the resynchronization of rhythms and stabilization of anxiety.

## Data availability statement

The original contributions presented in the study are included in the article/supplementary material, further inquiries can be directed to the corresponding author.

## Ethics statement

Ethical approval was not required for the study involving human samples in accordance with the local legislation and institutional requirements, because reason ethics approval was not required. Written informed consent for participation in this study was provided by the participants' legal guardians/next of kin. Written informed consent was obtained from the individual(s), and minor(s)' legal guardian/next of kin, for the publication of any potentially identifiable images or data included in this article.

## Author contributions

CE-D participated in the clinical treatment, wrote the article. EE and CE-D made the medical visits. BR-M processed with the ACM analytical data. JM assessed in the chronotherapy. All authors contributed to the article and approved the submitted version.
